# Deletion of Vaccinia Virus A40R Gene Improves the Immunogenicity of the HIV-1 Vaccine Candidate MVA-B

**DOI:** 10.3390/vaccines8010070

**Published:** 2020-02-06

**Authors:** Patricia Pérez, María Q. Marín, Adrián Lázaro-Frías, Carlos Óscar S. Sorzano, Carmen E. Gómez, Mariano Esteban, Juan García-Arriaza

**Affiliations:** 1 Department of Molecular and Cellular Biology, Centro Nacional de Biotecnología (CNB), Consejo Superior de Investigaciones Científicas (CSIC), 28049 Madrid, Spain; pperez@cnb.csic.es (P.P.); mquiros@cnb.csic.es (M.Q.M.); alazaro@cnb.csic.es (A.L.-F.); cegomez@cnb.csic.es (C.E.G.); mesteban@cnb.csic.es (M.E.); 2 Biocomputing Unit, Centro Nacional de Biotecnología (CNB), Consejo Superior de Investigaciones Científicas (CSIC), 28049 Madrid, Spain; coss@cnb.csic.es

**Keywords:** *A40R* gene, poxvirus, MVA, HIV vaccine, mice, immune responses

## Abstract

Development of a safe and efficacious vaccine against the HIV/AIDS pandemic remains a major scientific goal. We previously described an HIV/AIDS vaccine based on the modified vaccinia virus Ankara (MVA) expressing HIV-1 gp120 and Gag-Pol-Nef (GPN) of clade B (termed MVA-B), which showed moderate immunogenicity in phase I prophylactic and therapeutic clinical trials. Here, to improve the immunogenicity of MVA-B, we generated a novel recombinant virus, MVA-B ΔA40R, by deleting in the MVA-B genome the vaccinia virus (VACV) *A40R* gene, which encodes a protein with unknown immune function. The innate immune responses triggered by MVA-B ΔA40R in infected human macrophages, in comparison to parental MVA-B, revealed an increase in the mRNA expression levels of interferon (IFN)-β, IFN-induced genes, and chemokines. Compared to priming with DNA-B (a mixture of DNA-gp120 plus DNA-GPN) and boosting with MVA-B, mice immunized with a DNA-B/MVA-B ΔA40R regimen induced higher magnitude of adaptive and memory HIV-1-specific CD4+ and CD8+ T-cell immune responses that were highly polyfunctional, mainly directed against Env. and of an effector memory phenotype, together with enhanced levels of antibodies against HIV-1 gp120. Reintroduction of the A40R gene into the MVA-B ΔA40R genome (virus termed MVA-B ΔA40R-rev) promoted in infected cells high mRNA and protein A40 levels, with A40 protein localized in the cell membrane. MVA-B ΔA40R-rev significantly reduced mRNA levels of IFN-β and of several other innate immune-related genes in infected human macrophages. In immunized mice, MVA-B ΔA40R-rev reduced the magnitude of the HIV-1-specific CD4+ and CD8+ T cell responses compared to MVA-B ΔA40R. These results revealed an immunosuppressive role of the A40 protein, findings relevant for the optimization of poxvirus vectors as vaccines.

## 1. Introduction

The acquired immune deficiency syndrome (AIDS) pandemic caused by the human immunodeficiency virus (HIV)-1 is spreading worldwide, with high impact and severity in human health. In spite of active antiretroviral therapy (ART), in 2017, an estimated 1.8 million individuals became newly infected with HIV-1 and 940,000 people died from AIDS-related illnesses worldwide, according to the Joint United Nations Programme on HIV/AIDS. Therefore, the discovery of an effective vaccine against HIV/AIDS that could control the infection and disease progression should be one of the main priorities of the developed world.

An effective vaccine against HIV/AIDS should stimulate both humoral and cellular immune responses to multiple HIV-1 viral antigens, including structural and regulatory proteins, and induce strong, broad, polyfunctional, and durable T- and B-cell responses [1]. Although neutralizing antibodies against gp120 are crucial, due to the difficulty in obtaining immunogens capable of inducing high titers of neutralizing antibodies with broad specificities, a focus on HIV-1-specific T-cell immune responses has been one of the main routes pursued in the development of HIV-1 vaccines [2]. For example, in non-human primates, there is a good correlation between vaccine-induced HIV-1-specific cellular immunogenicity and protection after a challenge with a pathogenic simian/human immunodeficiency virus (SHIV) [3,4,5], where CD8+ T cells play an important role in immunity to HIV-1 [5]. Moreover, there is substantial evidence which points out that HIV-1-specific CD4+ and CD8+ T cells mediates protection in vivo [6], and the crucial role played by T cells in HIV-1 suppression comes from studying the immune system in “elite controllers”, a group of people who are able to control HIV-1 replication without any ART treatment [7,8]. Of the numerous clinical trials carried out so far with different HIV/AIDS vaccine candidates, only the RV144 phase III clinical trial showed a modest protection of 31.2% against HIV-1 infection. This clinical trial was based on priming with a recombinant canarypoxvirus ALVAC vector expressing the Env protein from subtypes B/E and Gag/Pro from subtype B, followed by boosting with HIV-1 gp120 protein from subtypes B/E [9]. Thus, improved poxvirus recombinants should be considered as components of an effective HIV/AIDS vaccine. One of the most promising poxvirus vectors is the modified vaccinia virus Ankara (MVA), which has been widely used as a vaccine candidate in preclinical and clinical trials against several prevalent and emerging infectious diseases, including HIV/AIDS, proving to be extremely safe, highly immunogenic, and protective [10,11,12,13,14,15].

Previously, we constructed a recombinant MVA expressing HIV-1 gp120 (engineered to be produced as a cell-released product) and Gag-Pol-Nef (GPN, as an intracellular polyprotein) antigens from clade B (termed MVA-B) [16]. MVA-B has been extensively studied in vitro and in different animal models [4,16,17,18,19,20,21,22,23,24,25]. Furthermore, MVA-B entered in a phase I clinical trial (RISVAC02) in healthy human volunteers, being well tolerated and eliciting moderate HIV-1-specific T-cell and antibody responses, mainly directed against the Env antigen, for almost one year [26,27]. Four years later, only 20% percent of vaccinees maintained low HIV-1-specific T-cell responses, suggesting that MVA-B lacks the capacity to induce long-term HIV-1-specific T-cell memory responses. However, a late MVA-B boost significantly increased the binding and neutralizing antibody responses in most of the vaccinees [28]. Moreover, in chronically HIV-1-infected individuals, vaccination with MVA-B enhanced HIV-1-specific CD4+ T cells but did not have a major impact on the latent reservoir or the rebound of plasma viral load after combined ART interruption [29,30,31,32]. After MVA-B therapeutic vaccination, a balance between activation and regulation of HIV-1-specific CD8+ T cell responses was observed [33], and in addition to inducing HIV-1-specific T-cell responses in chronically infected individuals, MVA-B has an effect on monocyte phenotype and their ability to produce cytokines [34]. Thus, optimized MVA-B vaccines that could further enhance the HIV-1-specific CD4+ and CD8+ T cell responses, as well as humoral responses, are most desirable.

There are several strategies available to enhance the magnitude, breadth, polyfunctionality, and durability of the immune responses to foreign antigens expressed from poxvirus vectors, such as (i) heterologous prime/boost immunization protocols, (ii) use of co-stimulatory molecules, (iii) deletion of vaccinia virus (VACV) immunomodulatory genes still present in the poxvirus genome, (iv) enhancing VACV promoter strength, (v) enhancing vector replication capacity, (vi) optimizing expression of foreign heterologous sequences, and (vii) combined use of adjuvants [35]. The MVA genome lacks multiple genes (about 30 kb) as a result of more than 570 passages in chick embryo fibroblast (CEF) cells [36,37,38]. However, MVA still contains several immunomodulatory genes that counteract the host antiviral innate immune response [39,40]. Hence, the deletion of these immunomodulatory genes is a promising approach for the generation of improved MVA-based vaccines [35]. We have previously described that deletions in the MVA-B genome of some immunomodulatory genes, such as *A41L*, *B16R*, *C6L*, *K7R*, and *N2L* (either as single deletions or in combination), have been associated with a significant enhancement in the magnitude, breadth, polyfunctionality, and durability of the HIV-1-specific T-cell and humoral immune responses in immunized mice, when compared to parental MVA-B [18,20,21,23]. Based on those results, novel optimized recombinant MVA vectors lacking the MVA immunosuppressive genes *C6L*, *K7R*, and *A46R* and expressing chikungunya virus [41], ebola virus [42], or zika virus [43] antigens have been generated, and were able to induce potent antigen-specific B- and T-cell immune responses in immunized mice and to protect against challenge with the corresponding viral pathogen in susceptible mouse models. 

MVA still contains several genes with unknown or suggested immunomodulatory functions, such as the *A40R* gene (*A40R* in the Copenhagen strain of VACV is equivalent to MVA152R in MVA. For simplicity, throughout this work, we used the open reading frame nomenclature of the Copenhagen strain to refer to the MVA genes), of which the role is controversial. Initial reports stated that this gene encodes a type II membrane glycoprotein (A40) with amino acid similarity (about 20%) to the complementarity-determining region (CDR) domain of C-type animal lectins such as the rat Clr-b, the NKG2 proteins, CD94, and DC-SIGN [44], a group of Ca^2+^-dependent (C-type) carbohydrate-binding (lectin) proteins that are very important in pathogen recognition and immunity [45]. This study also showed that VACV strain Western Reserve (WR) *A40R* gene product is expressed early during infection, forms high molecular mass complexes under non-reducing conditions, and is expressed on the cell surface but is not incorporated into intracellular mature virions (IMVs) or extracellularly enveloped virions (EEVs) [44]. Moreover, a deletion of the *A40R* gene attenuated VACV strain WR, following intradermal inoculation of mice, showing that A40 has a role in virulence [46]. Nevertheless, other studies affirmed that A40 is an early protein that is quantitatively sumoylated (a stable and infection-independent addition of the 20 kDa size peptide SUMO-1 which is mediated by cellular components) to prevent its own aggregation and allows the sumoylated protein to associate with the viral replication sites [47,48]. The small amount of non-modified A40 protein may play a putative role in the VACV life cycle, joining the cytosolic side of the rough endoplasmic reticulum (ER) and inducing the proper apposition of several ER cisternae before they fuse to generate the ER envelope that surround the viral replication sites [48]. However, the role of the sumoylated A40 protein still remains unclear, although it has been suggested that it could be involved in the process of replication itself or in the late transcription that is known to occur at VACV replication sites; in that case, the VACV *A40R* gene would be essential for the VACV life cycle. 

Thus, to define whether the VACV A40 protein has an immune function and to establish whether it might impact antigen-specific immune responses, we generated an MVA-B vector lacking the *A40R* gene (termed MVA-B ΔA40R), and examined the innate immune responses in cultured cells and the adaptive and memory HIV-1-specific T-cellular and humoral immune responses in immunized mice, in comparison to parental MVA-B. Moreover, we reintroduced the MVA *A40R* gene into the MVA-B ΔA40R genome (termed MVA-B ΔA40R-rev) and analyzed the mRNA and protein A40 levels, its localization in infected cells, and the impact in vitro on innate immune responses and on HIV-1 immunogenicity in vivo. Our results showed that MVA A40 protein has an immunosuppressive role, inhibiting innate immune responses in infected human macrophages, and revealed that deleting *A40R* in MVA-B enhances adaptive and memory HIV-1-specific T-cell and humoral immune responses. Therefore, deletion of the MVA *A40R* gene from poxvirus MVA vectors could be a good strategy to potentiate the vaccine immunogenicity of these vectors. 

## 2. Materials and Methods

### 2.1. Ethics Statement

Female Balb/cOlaHsd mice (6 to 8 weeks old) used for immunogenicity assays were purchased from Envigo Laboratories and stored in the animal facility of the CNB (Madrid, Spain). The immunogenicity animal studies were approved by the Ethical Committee of Animal Experimentation (CEEA) of the CNB (Madrid, Spain) and by the Division of Animal Protection of the Comunidad de Madrid (PROEX 331/14). All animal procedures were conformed to international guidelines and to the Spanish law under the Royal Decree (RD 53/2013). 

### 2.2. Cells

DF-1 cells (a spontaneously immortalized chicken embryo fibroblast (CEF) cell line, ATCC catalog no. CRL-12203), primary CEF cells (obtained from specific-pathogen-free 11 day old eggs; MSD, Salamanca, Spain), and HeLa cells (immortalized human epithelial cervix adenocarcinoma cells, ATCC^®^ CCL-2) were grown in Dulbecco’s modified Eagle’s medium (DMEM, Gibco-Life Technologies, Carlsbad, CA, USA) supplemented with 10% fetal calf serum (FCS, Gibco-Life Technologies, Carlsbad, CA, USA) for DF-1 and CEF cells or 10% newborn calf serum (NCS, Sigma-Aldrich, St. Louis, MO, USA) for HeLa cells. The THP-1 human monocytic cell line (ATCC catalog no. TIB-202) was cultured in Roswell Park Memorial Institute (RPMI) 1640 (Gibco-Life Technologies, Carlsbad, CA, USA) medium supplemented with HEPES pH 7.4 (10 mM, Merck, Darmstadt, Germany), β-mercaptoethanol (10 µM, Sigma-Aldrich, St. Louis, MO, USA), L-glutamine (2 mM, Merck, Darmstadt, Germany), and 10% FCS. THP-1 cells were differentiated into macrophages by treatment with 0.5 mM phorbol 12-myristate 13-acetate (Sigma-Aldrich, St. Louis, MO, USA) for 24 h before use. Cell cultures were maintained at 37 °C in a humidified incubator containing 5% CO_2_. Cell lines were infected with viruses and, after 1 h of adsorption, the virus inoculum was removed and DMEM-2% FCS or DMEM-2% NCS was added to the cell cultures. 

### 2.3. Viruses

The viruses used in this study included the attenuated MVA wild-type (MVA-WT) strain (kindly provided by G. Sutter) obtained from the Chorioallantois vaccinia virus Ankara (CVA) strain after 586 serial passages in CEF cells [38], and the recombinant MVA-B expressing HIV-1_IIIB_ GPN as an intracellular polyprotein and the HIV-1_BX08_ gp120 protein as a cell-released product from HIV-1 clade B isolates [16], which are inserted into the TK locus of the MVA-WT genome under the transcriptional control of a VACV sE/L promoter. MVA-B was used as the parental virus for the generation of the MVA-B ΔA40R deletion mutant, and MVA-B ΔA40R was used as the parental virus for the generation of the MVA-B ΔA40R-rev virus. All viruses were grown in DF-1 cells to obtain a master seed stock (P2 stock), and titrated in DF-1 cells by plaque immunostaining, using rabbit polyclonal antibody against VACV strain WR (CNB; diluted 1:1000), followed by an anti-rabbit horseradish peroxidase (HRP)-conjugated secondary antibody (Sigma-Aldrich, St. Louis, MO, USA; diluted 1:1000), as previously described [49]. Determinations of the titers of the different viruses were performed at least two times. Furthermore, viruses grown in primary CEF cells were purified by centrifugation through two 36% (wt./vol.) sucrose cushions in 10 mM Tris-HCl pH 9. All viral stocks were free of contamination with mycoplasma (checked by specific polymerase chain reaction (PCR) for mycoplasma), bacteria (checked by growth in LB plates without ampicillin), or fungi (checked by growth in Columbia blood agar plates; Oxoid, Hampshire, UK).

### 2.4. Plasmids

#### 2.4.1. Construction of Plasmid Transfer Vector pGem-RG-ΔA40R wm 

This plasmid was used for the construction of the MVA-B ΔA40R deletion mutant from which the MVA *A40R* gene has been deleted (*A40R* in the Copenhagen strain of VACV is equivalent to MVA152R in MVA. For simplicity, throughout this work, we have used the open reading frame nomenclature of the Copenhagen strain to refer to the MVA genes). The plasmid transfer vector pGem-RG-ΔA40R wm (“wm” stands for “without markers”, indicating that the final recombinant virus lacks the markers used for its selection, dsRed2 and rsGFP) was obtained by sequential cloning of MVA *A40R* flanking sequences into plasmid pGem-RG wm (4540 bp), the generation of which has been previously described [21], and contains the genes for dsRed2 and red-shifted green fluorescent protein (rsGFP) fluorescent markers. The MVA-B genome was used as the template to amplify by PCR the left flank of the *A40R* gene (352 bp) with oligonucleotides LFA40R-AatII-F (5′-ACGTTTGACGTCATAGAAAAATATAA-3′) (AatII site underlined) and LFA40R-XbaI-R (5′-TACACCGCACGACAATGAACAAACAT-3′) (XbaI site underlined). The left flank was digested with AatII and XbaI and cloned into plasmid pGem-RG wm, which had previously been digested with the same restriction enzymes to generate pGem-RG-LFsA40R wm (4859 bp). The repeated left flank of the *A40R* gene (352 bp) was amplified by PCR from the MVA-B genome with oligonucleotides LF′A40R-EcoRI-F (5′-ACGTTTGAATTCATAGAAAAATATAA-3′) (EcoRI site underlined) and LF′A40R-ClaI-R (5′-TACACCGCACGACAATGAACAAACAT-3′) (ClaI site underlined), digested with EcoRI and ClaI, and inserted into EcoRI/ClaI-digested pGem-RG-LFsA40R wm to generate plasmid pGem-RG-LFdA40R wm (5170 bp). Finally, the right flank of the *A40R* gene (372 bp) was amplified by PCR from the MVA-B genome with oligonucleotides RFA40R-ClaI-F (5′-AGAAAAATCGATATATCGCCGTACCG-3′) (ClaI site underlined) and RFA40R-BamHI-R (5′-CTGTTAATTTTACTAGATCGTCAT GG-3′) (BamHI site underlined), digested with ClaI and BamHI, and inserted into ClaI/BamHI-digested pGem-RG-LFdA40R wm plasmid. The resulting plasmid transfer vector generated was termed pGem-RG-ΔA40R wm (5512 bp), and directs the deletion of the *A40R* gene from the MVA-B genome. Its correct construction was confirmed by DNA sequence analysis.

#### 2.4.2. Construction of Plasmid Transfer Vector pHA-A40R

This plasmid was used for the insertion of the MVA *A40R* gene into the MVA hemagglutinin (HA) locus of the MVA-B ΔA40R recombinant virus to generate the MVA-B ΔA40R-rev revertant virus. To construct the plasmid transfer vector pHA-A40R (7126 bp), the MVA *A40R* gene (526 bp) was amplified by PCR from the MVA-B genome with oligonucleotides LFA40R-XmaI-F (5′-TCCCCCCGGGATGAACAAACATAAGAC-3′) (XmaI site underlined) and RFA40R-SacII-R (5′-AGGCCGCGGTTATTTTTTTCTAAAACACTC-3′) (SacII site underlined), digested with XmaI and SacII restriction enzymes, and then inserted into the XmaI/SacII-digested pHA plasmid (6600 bp), the generation of which has been previously described [42]. Thus, pHA-A40R contained the MVA *A40R* gene under the control of the VACV sE/L promoter introduced in a multiple-cloning site between the MVA HA-L and HA-R flanking regions, and the selectable marker genes for ampicillin and β-glucuronidase (β-gus). The β-gus gene was inserted among two repetitions of the left HA flanking region, allowing their deletion from the final recombinant virus by homologous recombination after consecutive plaque purification steps. The correct construction of plasmid pHA-A40R was confirmed by DNA sequence analysis.

### 2.5. Generation of Recombinant MVA-B Δ40R and MVA-B ΔA40R-rev Viruses

The MVA-B ΔA40R deletion mutant contained a deletion of the MVA *A40R* gene in the MVA-B genome and the MVA-B ΔA40R-rev revertant virus contained an insertion of the MVA *A40R* gene in the MVA HA locus of MVA-B ΔA40R. MVA-B ΔA40R and MVA-B ΔA40R-rev were generated using MVA-B and MVA-B ΔA40R as parental viruses, respectively, and pGem-RG-ΔA40R wm and pHA-A40R as plasmid transfer vectors, respectively, employing an infection/transfection protocol previously described [18,20,23,42,43,50]. In the case of MVA-B ΔA40R, after the infection/transfection in DF-1 cells, we selected plaques expressing both Red2/GFP fluorescent proteins, then plaques expressing only GFP, and lastly we selected viruses from plaques that did not express any marker due to the loss of the fluorescent marker, as previously described [18,21]. Thus, MVA-B ΔA40R was finally obtained after six consecutive rounds of plaque purification. In the case of MVA-B ΔA40R-rev, after the infection/transfection in DF-1 cells, we initially selected blue plaques stained with 5-bromo-4-chloro-3-indolyl-beta-d-glucuronic acid (β-Gus, Sigma-Aldrich, St. Louis, MO, USA). In the first three passages viruses from selected blue plaques expressing β-Gus were picked, and in the last three passages (six passages in total) viruses from selected plaques did not express any marker due to the loss of the β-Gus marker. In both cases, the isolated plaques were expanded in DF-1 cells until a cytopathic effect was observed, and then crude viral extracts obtained were used for the next plaque purification round. After these recombination events in cell culture, the final plaques of both recombinant viruses, MVA-B ΔA40R and MVA-B ΔA40R-rev, were selected and expanded in DF-1 cells to obtain a master seed stock (P2 stock). 

### 2.6. PCR Analysis

To verify that the MVA *A40R* gene had been correctly deleted in MVA-B ΔA40R or correctly inserted in MVA-B ΔA40R-rev, viral DNA was extracted from DF-1 cells mock infected or infected at 5 PFU/cell with the different viruses, as previously described [43], and the correct deletion or insertion of the MVA *A40R* gene was confirmed by PCR analysis. Primers LFA40R-AatII-F and RFA40R-BamHI-R (described above), spanning the *A40R* flanking regions, were used for PCR analysis of the *A40R* locus to verify the correct deletion of the MVA gene *A40R* in MVA-B ΔA40R, while primers HA-2 (5′-GATCCGCATCATCGGTGG-3′) and HA-MVA (5′-TGACACGATTACCAATAC-3′), annealing in the MVA HA gene-flanking regions, were used for PCR analysis of the MVA HA locus, to verify the correct insertion of the MVA gene *A40R* in MVA-B ΔA40R-rev. The *A40R* deletion and insertion were also confirmed by DNA sequence analysis (Secugen, Madrid, Spain). The amplification protocols were performed using PuReTaq™ Ready-To-Go™ PCR beads (GE Healthcare, Chicago, IL, USA), in accordance with the manufacturer’s protocol. PCR products were run in 1% agarose gel and visualized by SYBR Safe staining (Invitrogen, Carlsbad, CA, USA).

### 2.7. Expression of HIV-1_BX08_ gp120 and HIV-1_IIIB_ GPN Proteins by Western Blot

To check the correct expression of the HIV-1_BX08_ gp120 and HIV-1_IIIB_ GPN proteins, monolayers of DF-1 cells were mock infected or infected at 5 plaque forming units (PFU)/cell with the different viruses. At 24 h.p.i., cell extracts were lysed in Laemmli buffer and fractionated in 8% SDS-PAGE, and then analyzed by Western blotting with rabbit polyclonal anti-gp120 antibody against IIIB (CNB; diluted 1:3,000) or polyclonal anti-gag p24 serum (ARP 432, diluted 1:1000; National Institute for Biological Standards and Control (NIBSC) Centre for AIDS Reagents) to analyze the expression of the gp120 and GPN proteins, respectively. As loading controls, we used rabbit anti-α-tubulin (Cell Signaling, Danvers, MA, USA, diluted 1:1000), rabbit anti-β-actin (Cell Signaling, Danvers, MA, USA; diluted 1:1000), and rabbit anti-VACV E3 (CNB; diluted 1:1000) antibodies. An HRP-conjugated anti-rabbit antibody (Sigma-Aldrich, St. Louis, MO, USA; diluted 1:5000) was used as the secondary antibody. The immunocomplexes were detected with an HRP-luminol enhanced-chemiluminescence system (ECL Plus) (GE Healthcare, Chicago, IL, USA).

### 2.8. Analysis of Virus Growth

To study the virus growth profile of MVA-B, MVA-B ΔA40R, and MVA-B ΔA40R-rev, monolayers of DF-1 cells grown in 12 well plates were infected in duplicate at 0.01 PFU/cell with the different viruses. Following virus adsorption for 60 min at 37 °C, the inoculum was removed. The infected cells were washed with DMEM and incubated with fresh DMEM containing 2% FCS at 37 °C in a 5% CO_2_ atmosphere. At different times (0, 24, 48, and 72 h.p.i.), cells were collected by scraping, freeze–thawed three times, and briefly sonicated. Virus titers in cell lysates were determined by immunostaining plaque assay as previously described [49].

### 2.9. RNA Analysis by Reverse Transcription Real-Time Quantitative PCR (RT-qPCR)

Total RNA was isolated using the RNeasy Kit (Qiagen, Hilden, Germany), from THP-1 cells mock infected or infected at 5 PFU/cell with the different viruses, and harvested at 3 and/or 6 h.p.i. Reverse transcription of maximum 1000 ng of RNA was performed with the QuantiTect reverse transcription kit (Qiagen, Hilden, Germany), according to the manufacturer’s recommendations. Quantitative PCR was performed with a 7500 Real-Time PCR system (Applied Biosystems, Foster City, CA, USA) using Power SYBR green PCR Master Mix (Applied Biosystems, Foster City, CA, USA), as previously described [51]. The mRNA expression levels of the genes for IFN-β, interferon-induced protein with tetratricopeptide repeats 1 (IFIT1), IFIT2, melanoma differentiation-associated protein 5 (MDA-5), retinoic acid-inducible gene I (RIG-I), tumour necrosis factor alpha (TNF-α), regulated on activation, normal T cell expressed and secreted (RANTES), and macrophage inflammatory protein 1 alpha (MIP-1α) were analyzed by real-time PCR with specific oligonucleotides (sequences are available upon request). Specific gene expression was expressed relative to the expression of the cellular hypoxanthine phosphoribosyltransferase (HPRT) gene and/or the VACV *E3L* gene in arbitrary units (A.U.) using the 2^−ΔΔCt^ method [52]. All samples were tested in triplicate, and two independent experiments were performed. 

### 2.10. DNA Vectors

DNA plasmids expressing HIV-1_BX08_ gp120 (pCMV-_BX08_gp120) and HIV-1_IIIB_ GPN fusion protein (pcDNA-_IIIB_GPN) and the empty plasmid (pcDNA-ϕ) have been previously described [16], and were purified with the EndoFree Plasmid Mega kit (Qiagen, Hilden, Germany) in accordance with the manufacturer’s protocol and diluted for injection in endotoxin-free phosphate-buffered saline (PBS) (Gibco-Life Technologies, Carlsbad, CA, USA). pcDNA-ϕ (termed DNA-ϕ) or a mixture of pCMV-_BX08_gp120 and pcDNA-_IIIB_GPN (termed DNA-B) were used in the immunization protocol as a prime.

### 2.11. Peptides

HIV-1 peptide pools, with each purified peptide at 1 mg/mL per vial, were provided by EuroVacc. The peptides covered the Env, Gag, Pol, and Nef proteins present in the consensus sequence of HIV-1 clade B (gp120 from isolate BX08 and GPN from isolate IIIB) as consecutive 15-mers overlapping by 11 amino acids. The HIV-1_BX08_ gp120 protein was spanned by the Env-1 and Env-2 peptide pools. The HIV-1_IIIB_ GPN fusion protein was spanned by the Gag-1, Gag-2, GPN-1, GPN-2, GPN-3, and GPN-4 pools. For immunological analysis, we grouped the peptides into three main pools, Env, Gag, and GPN. The Env pool comprised Env-1 and Env-2, the Gag pool comprised Gag-1 and Gag-2, and the GPN pool comprised GPN-1, GPN-2, GPN-3, and GPN-4. 

### 2.12. Mouse Immunization Schedule

Female BALB/c mice (6 to 8 weeks old) were purchased from Envigo Laboratories and stored in a pathogen-free barrier area of the CNB in accordance with the recommendations of the Federation of European Laboratory Animal Science Associations. A DNA prime/MVA boost immunization protocol was performed as previously described [16,18,20,21,23] to assay the immunogenicity of MVA-WT, MVA-B, MVA-B ΔA40R, and MVA-B ΔA40R-rev. Groups of animals (n = 8) received 100 μg of DNA-B (a mixture of 50 μg of pCMV-_BX08_gp120 plus 50 μg of pCDNA-_IIIB_GPN) or 100 μg of DNA-ϕ (100 μg of pcDNA-ϕ) by the i.m. route and 2 weeks later received an i.p. inoculation of 1 × 10^7^ PFU of the corresponding MVA virus (MVA-WT, MVA-B, MVA-B ΔA40R, or MVA-B ΔA40R-rev) in 200 μL of PBS. Mice primed with sham DNA (DNA-ϕ) and boosted with non-recombinant MVA-WT were used as a control group. At 10 and 53 days after the last immunization, four mice in each group were sacrificed via carbon dioxide (CO_2_) and their spleens were processed to measure the adaptive and memory immune responses to HIV-1 antigens, respectively, by intracellular cytokine staining (ICS) assay. Three independent experiments were performed. A MVA prime/MVA boost immunization protocol was also performed to further assay the immunogenicity of MVA-WT, MVA-B, MVA-B ΔA40R, and MVA-B ΔA40R-rev, in four mice receiving two i.p. inoculations of 2 × 10^7^ PFU of the corresponding MVA virus (MVA-WT, MVA-B, MVA-B ΔA40R, or MVA-B ΔA40R-rev). Ten days after the last immunization, mice were sacrificed via carbon dioxide (CO_2_) and their spleens were processed to measure the adaptive immune responses to HIV-1 antigens by ICS assay.

### 2.13. ICS Assay

The magnitude, breadth, polyfunctionality, and phenotypes of the HIV-1-specific T-cell adaptive and memory responses were analyzed by ICS as previously described [18,20,23,43,53], with some modifications. After spleen processing, 4 × 10^6^ fresh splenocytes (depleted of red blood cells) were seeded onto M96 plates and stimulated for 6 h in complete RPMI 1640 medium supplemented with 10% FCS containing 1 μL/ml Golgiplug (BD Biosciences, Franklin Lakes, NJ, USA) to inhibit cytokine secretion; monensin 1X (eBioscience, Thermo Fisher Scientific, Waltham, MA, USA), anti-CD107a–FITC (BD Biosciences, Franklin Lakes, NJ, USA); and the different HIV-1 Env, Gag, and GPN pools of peptides (5 μg/mL). Cells were then washed, stained for surface markers, fixed, permeabilized (Cytofix/Cytoperm kit; BD Biosciences, Franklin Lakes, NJ, USA), and stained intracellularly with the appropriate fluorochromes. Dead cells were excluded using the violet LIVE/DEAD stain kit (Invitrogen, Carlsbad, CA, USA). The fluorochrome-conjugated antibodies used for functional analyses were CD3-phycoerythrin (PE)-CF594, CD4-allophycocyanin (APC)-Cy7, CD8-V500, IFN-γ–PE-Cy7, TNF-α–PE, and IL-2–APC. In addition, the antibodies used for phenotypic analyses were CD62L-Alexa 700 and CD127-peridinin chlorophyll protein (PerCP)-Cy5.5. All antibodies were from BD Biosciences. Cells were acquired with a Gallios flow cytometer (Beckman Coulter). Analyses of the data were performed with the FlowJo software version 8.5.3 (Tree Star, Ashland OR, USA). After gating, Boolean combinations of single functional gates were created using FlowJo software to determine the frequency of each response based on all possible combinations of cytokine expression or all possible combinations of differentiation marker expression. Background responses detected in negative control samples were subtracted from those detected in stimulated samples for every specific functional combination.

### 2.14. Antibody Measurements by Enzyme-Linked Immunosorbent Assay (ELISA)

The total IgG, IgG1, IgG2a, and IgG3 anti-HIV-1 gp120 BX08 envelope protein antibodies in pooled sera from immunized mice were measured by ELISA, as previously described [16,21].

### 2.15. Statistical Procedures

For statistical analysis of cytokine/chemokine expression, one-way analysis of variance (ANOVA) with Tukey’s honestly significant difference (HSD) post hoc tests were applied. Student’s *t*-test was used for antibody analysis to establish the differences between two groups. Statistical analysis of the ICS assay results was realized as previously described [21,53], using an approach that corrects measurements for the medium response (RPMI), calculating confidence intervals and *p* values. Only antigen response values significantly larger than the corresponding RPMI are presented. Background values were subtracted from all of the values used to allow analysis of proportionate representation of responses. The statistical significances are indicated as follows: *, *p* < 0.05; **, *p* < 0.005; ***, *p* < 0.001. 

## 3. Results

### 3.1. Generation and In Vitro Characterization of MVA-B ΔA40R

**Figure 1:**
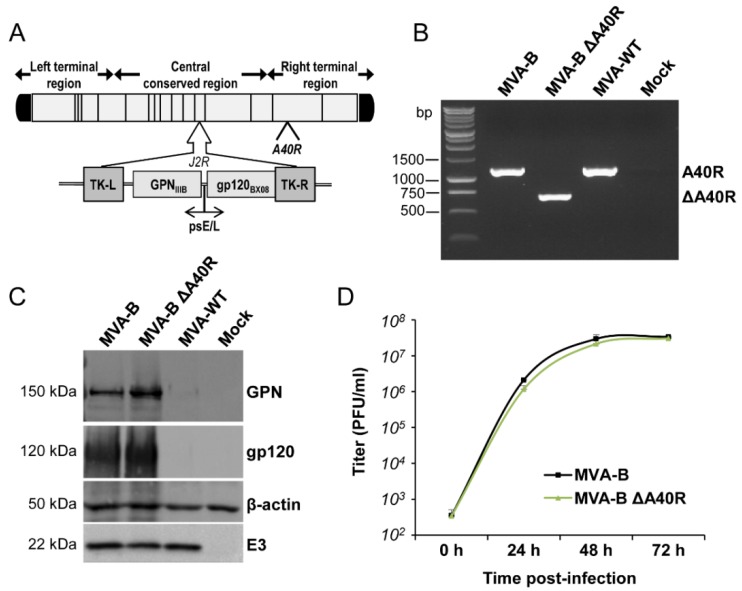
Generation and *in vitro* characterization of MVA-B ΔA40R. (**A**) Scheme of the MVA-B ΔA40R genome map (adapted from References [37,54]). The central conserved region and the left and right terminal regions are shown. Below the map, the deleted or fragmented genes are depicted as black boxes. The deleted *A40R* gene is indicated. The HIV-1 GPN (from isolate IIIB) and gp120 (from isolate Bx08) clade B sequences driven by the VACV sE/L promoter (psE/L) inserted within the TK viral locus (*J2R*) are indicated. TK-L = TK left flanking region, TK-R = TK right flanking region. (**B**) PCR analysis of the *A40R* locus. Viral DNA was extracted from DF-1 cells mock infected or infected at 5 PFU/cell with MVA-WT, MVA-B, or MVA-B ΔA40R. Primers spanning *A40R* flanking regions were used for PCR analysis of the *A40R* locus. DNA products corresponding to the *A40R* gene and the *A40R* deletion are indicated on the right. Molecular size markers (1 kb ladder) with the corresponding sizes (base pairs) are indicated on the left. (**C**) Expression of HIV-1_BX08_ gp120 and HIV-1_IIIB_GPN proteins. DF-1 cells were mock infected or infected at 5 PFU/cell with MVA-WT, MVA-B, or MVA-B ΔA40R. At 24 h.p.i., cells were lysed in Laemmli buffer, fractionated by 8% SDS-PAGE, and analyzed by Western blotting with rabbit polyclonal anti-gp120 antibody or polyclonal anti-gag p24 serum. Rabbit anti-β-actin and rabbit anti-VACV early E3 protein antibodies were used as protein and VACV loading controls, respectively. The proteins detected are indicated on the right and their protein molecular weights (in kDa) are indicated on the left. (**D**) Viral growth kinetics. DF-1 cells were infected at 0.01 PFU/cell with MVA-B or MVA-B ΔA40R. At different times (0, 24, 48, and 72 h.p.i.) cells were collected and virus titers of cell lysates were quantified via plaque immunostaining assay with anti-VACV antibodies. The mean and standard deviation of two independent experiments is shown.

To determine whether the MVA *A40R* gene might have an immunomodulatory role that, in turn, could influence the immunogenicity profile of antigens delivered from a poxvirus vector, we deleted the MVA *A40R* gene from the HIV/AIDS vaccine candidate MVA-B (expressing HIV-1 Env, Gag, Pol, and Nef antigens from clade B) [16], generating the MVA-B deletion mutant termed MVA-B ΔA40R (see Materials and Methods) (Figure 1A). The presence of the *A40R* deletion was confirmed by PCR of the MVA *A40R* viral locus (Figure 1B), and was also validated by DNA sequencing. Analysis by Western blotting demonstrated that MVA-B ΔA40R expressed HIV-1_BX08_ gp120 and HIV-1_IIIB_ GPN antigens similarly as the parental MVA-B (Figure 1C). 

### 3.2. A40 Was Non-Essential in Cell Culture

The mere isolation of MVA-B ΔA40R confirmed that the A40 protein is not essential for MVA replication. Next, to examine whether deletion of *A40R* altered virus multiplication, we compared the growth of MVA-B ΔA40R and MVA-B in cultured permissive DF-1 cells. The growth kinetics of parental MVA-B and deletion mutant MVA-B ΔA40R were similar (Figure 1D), confirming that the MVA A40 protein is not required for MVA replication.

### 3.3. Deletion of MVA A40R Gene Enhanced the MVA-B Innate Immune Responses in Human Macrophages

**Figure 2:**
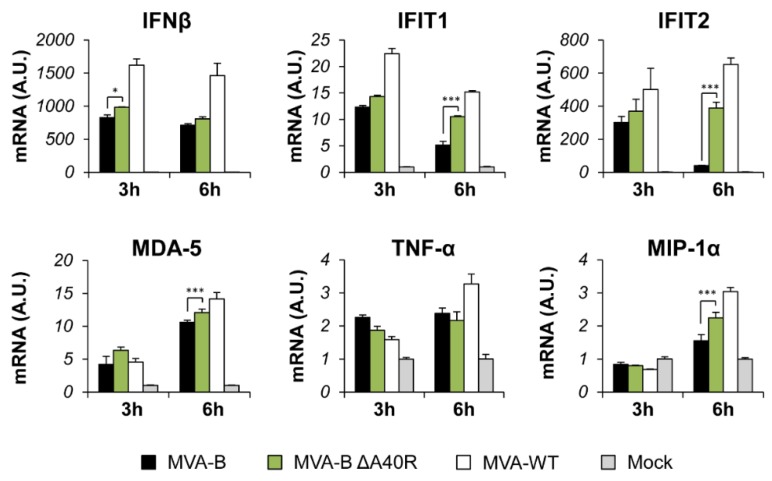
MVA-B ΔA40R upregulated the levels of type I IFN, proinflammatory cytokines, and chemokine expression compared to parental MVA-B. Human THP-1 macrophages were mock infected or infected with MVA-WT, MVA-B, or MVA-B ΔA40R at 5 PFU/cell. At 3 and 6 h.p.i., RNA was extracted, and IFN-β, IFIT1, IFIT2, MDA-5, TNF-α, MIP-1α, and HPRT mRNA levels were analyzed by RT-qPCR. Results are expressed as the ratio of the gene of interest to HPRT mRNA levels. A.U., arbitrary units. *p* values indicate significant response differences between MVA-B and MVA-B ΔA40R at the same hour (*, *p* < 0.05; ***, *p* < 0.001). Data shown are means ± standard deviations of triplicate samples from one experiment and are representative of two independent experiments.

The production of type I IFN, pro-inflammatory cytokines, and chemokines is an important initial step in the induction of antiviral immunity [55,56]. Thus, to study whether MVA A40 protein impacts innate immune responses, human THP-1 macrophages were mock infected or infected for 3 and 6 h with MVA-WT, MVA-B, and MVA-B ΔA40R at 5 PFU/cell, and the mRNA expression levels of type I IFN (IFN-β), type I IFN-induced genes (IFIT1 and IFIT2), the viral dsRNA sensor MDA-5, the proinflammatory cytokine TNF-α, and the chemokine MIP-1α were analyzed by reverse transcription real-time quantitative PCR (RT-qPCR). The results showed that, compared to parental MVA-B, MVA-B ΔA40R significantly upregulated the mRNA levels of IFN-β, IFIT1, IFIT2, MDA-5, and MIP-1α, at 3 or 6 h, but did not affect the mRNA expression of TNF-α (Figure 2), suggesting an immunosuppressive function of the MVA A40 protein. 

### 3.4. MVA-B ΔA40R Increased the Magnitude of HIV-1-Specific T-Cell Adaptive Immune Responses

Given the apparent immunosuppressive role of the MVA A40 protein in impairing the innate immune responses in human macrophages, we next asked whether deletion of MVA *A40R* from MVA-B could have an impact on the immunogenicity of the vector. Therefore, to study in vivo the effect of the *A40R* deletion on the HIV-1-specific T-cellular immunogenicity elicited by the HIV/AIDS vaccine candidate MVA-B, we analyzed the HIV-1-specific CD4+ and CD8+ T-cell immune responses induced by MVA-B ΔA40R in Balb/c mice immunized with a DNA prime/MVA boost immunization regimen, as this protocol amplifies the levels of T- and B-cell responses, while the homologous MVA prime/MVA boost immunization triggers lower responses [4,16]. Mice received 100 μg of DNA-B prime (50 μg of DNA-GPN plus 50 μg of DNA-gp120) by intramuscular (i.m.) route, and 14 days later were boosted with 1 × 10^7^ plaque-forming units (PFUs) of MVA-B or MVA-B ΔA40R viruses by intraperitoneal (i.p) route. Animals primed with sham DNA (DNA-ϕ) and boosted with non-recombinant MVA-WT were used as a control group. Adaptive HIV-1-specific CD4+ and CD8+ T cell immune responses elicited by the different immunization groups (DNA-B/MVA-B, DNA-B/MVA-B ΔA40R, and DNA-ϕ/MVA-WT) were measured 10 days post-boost by intracellular cytokine staining (ICS) assay, after the stimulation of splenocytes with pools of peptides (Env, Gag, and GPN peptide pools) that spanned the HIV-1 Env, Gag, Pol, and Nef antigens from an HIV-1 clade B consensus sequence. 

**Figure 3:**
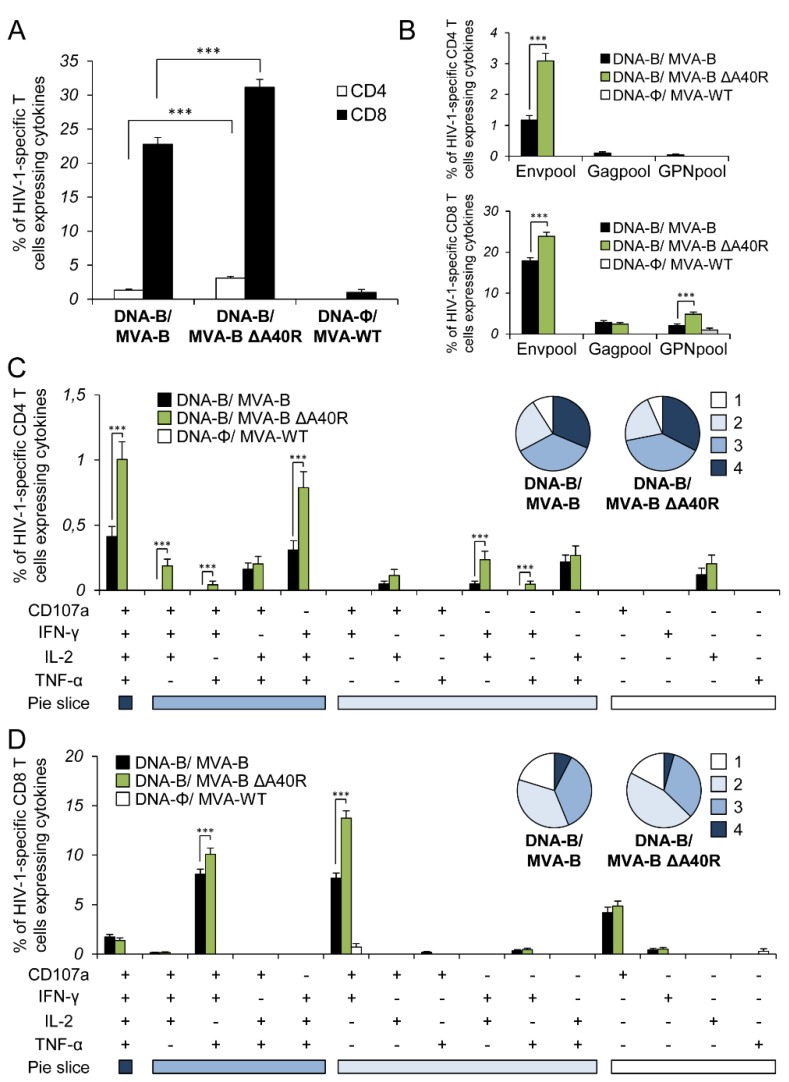
Immunization with MVA-B ΔA40R enhanced the magnitude of HIV-1-specific CD4+ and CD8+ T-cell adaptive immune responses. Splenocytes were collected from mice (n = 4 per group) immunized with DNA-ϕ/MVA-WT, DNA-B/MVA-B or DNA-B/MVA-B ΔA40R 10 days after the last immunization. Next, HIV-1-specific CD4+ and CD8+ T-cell adaptive immune responses triggered by the different immunization groups were measured by ICS assay following the stimulation of splenocytes with different HIV-1 peptide pools (Env, Gag, and GPN). Values from unstimulated controls were subtracted in all cases. *p* values indicate significant response differences between the DNA-B/MVA-B ΔA40R and DNA-B/MVA-B immunization groups (***, *p* < 0.001). Data are from one experiment representative of three independent experiments. (**A**) Overall percentages of HIV-1-specific CD4+ and CD8+ T cells. The values represent the sum of the percentages of T cells expressing CD107a and/or IFN-γ and/or TNF-α and/or IL-2 against Env, Gag, and GPN peptide pools. (**B**) Percentages of Env, Gag, and GPN HIV-1-specific CD4+ and CD8+ T cells. Frequencies represent the sum of the percentages of T cells expressing CD107a and/or IFN-γ and/or TNF-α and/or IL-2 against Env, Gag, or GPN peptide pools. (**C**,**D**) Polyfunctional profiles of HIV-1-specific CD4+ (**C**) and CD8+ (**D**) T cells. All of the possible combinations of responses are shown on the x axis, while the percentages of T cells expressing CD107a and/or IFN-γ and/or TNF-α and/or IL-2 against Env, Gag, and GPN peptide pools are shown on the y axis. Responses are grouped and color-coded on the basis of the number of functions (4, 3, 2, or 1). The pie charts summarize the data. Each slice corresponds to the proportion of the total HIV-1-specific CD4+ and CD8+ T cells exhibiting 1, 2, 3, or 4 functions (CD107a and/or IFN-γ and/or TNF-α and/or IL-2) within the total HIV-1-specific CD4+ and CD8+ T cells.

The magnitude of the total HIV-1-specific CD4+ and CD8+ T-cell adaptive immune responses (determined as the sum of the individual responses producing IFN-γ, TNF-α, and/or IL-2 cytokines, as well as the expression of CD107a on the surface of activated T cells as an indirect marker of cytotoxicity; obtained for the Env, Gag, and GPN peptide pools) was significantly greater in the DNA-B/MVA-B ΔA40R immunization group than in DNA-B/MVA-B (2.3- and 1.4-fold times higher, respectively), with both vaccinated groups triggering an overall HIV-1-specific immune response mediated mainly by CD8+ T cells (91% and 95%, respectively) (Figure 3A). 

The pattern of HIV-1-specific T-cell adaptive immune responses showed that CD4+ and CD8+ T cell responses were directed mainly against the Env pool in both vaccinated groups, with CD8+ T cell responses broadly distributed among Env, Gag, and GPN (Figure 3B). However, DNA-B/MVA-B ΔA40R significantly enhanced the magnitude of Env-specific CD4+ T cell responses and Env- and GPN-specific CD8+ T cell responses (Figure 3B).

Furthermore, the quality of the HIV-1-specific T-cell adaptive immune response was characterized in part by the pattern of cytokine production and its cytotoxic potential. On the basis of the production of CD107a, IFN-γ, TNF-α, and IL-2 from HIV-1-specific CD4+ and CD8+ T cells, 15 different HIV-1-specific CD4+ and CD8+ T cell populations could be identified (Figure 3C,D). As shown in Figure 3C (pie charts), HIV-1-specific CD4+ T-cell responses were similarly polyfunctional in both vaccinated groups, with around 90% of the CD4+ T cells exhibiting two or more functions. CD4+ T cells expressing CD107a-IFN-γ-TNF-α-IL-2, and IFN-γ-TNF-α-IL-2 were the most induced populations elicited by both vaccinated groups, with DNA-B/MVA-B ΔA40R inducing a significantly greater percentage of most of the CD4+ T cells exhibiting four, three, two, or one functions than DNA-B/MVA-B (Figure 3C, bars). On another side, as shown in Figure 3D (pie charts), DNA-B/MVA-B and DNA-B/MVA-B ΔA40R had a similar polyfunctionality profile of HIV-1-specific CD8+ T-cell responses, with 80% and 83% of the CD8+ T cells exhibiting two or more functions, respectively. CD8+ T cells expressing CD107a-IFN-γ-TNF-α, and CD107a-IFN-γ were the most abundant populations elicited by both vaccinated groups, with DNA-B/MVA-B ΔA40R inducing, once again, a significantly greater increase in the percentage of those populations than DNA-B/MVA-B (Figure 3D, bars). 

### 3.5. MVA-B ΔA40R Improved HIV-1-Specific T-Cell Memory Immune Responses

Memory T cell responses might be critical for protection against HIV-1 infection [57,58,59,60], and the durability of a vaccine-induced T-cell response is an important feature, since long-term protection is a requirement for prophylactic vaccination. Thus, we next analyzed the HIV-1-specific T-cell memory immune responses elicited by the different immunization groups 53 days after the boost, following the same ICS assay described in the adaptive phase. 

**Figure 4:**
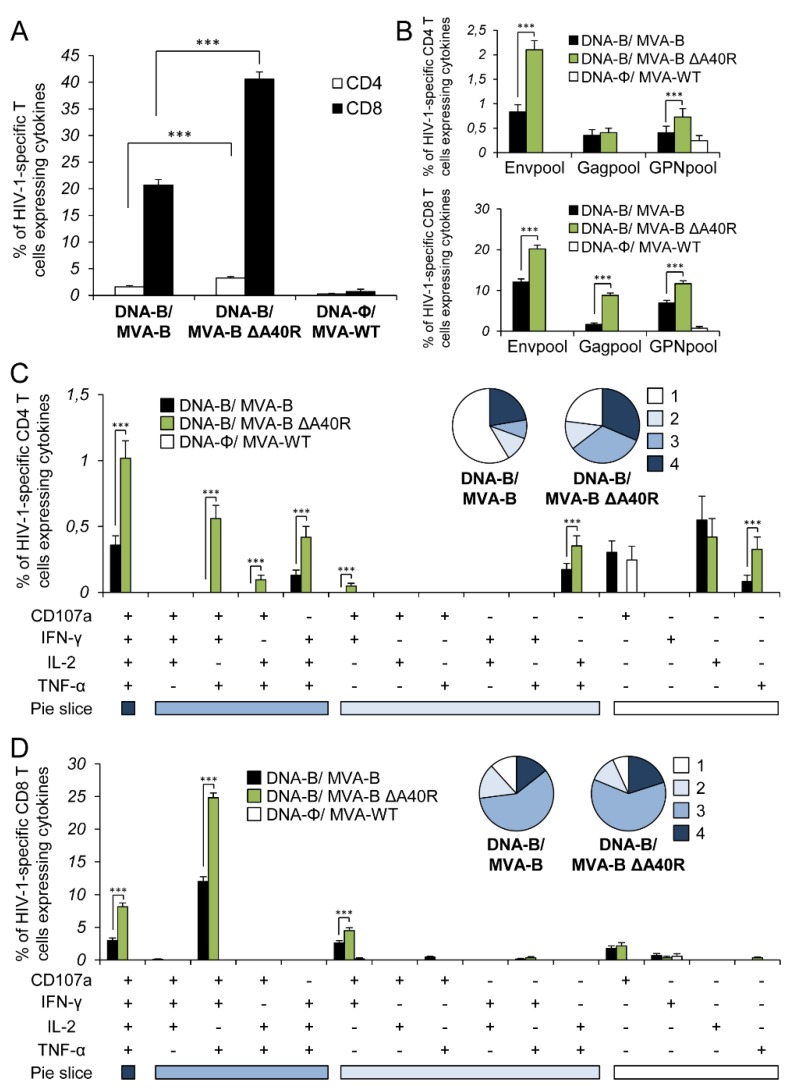
Immunization with MVA-B ΔA40R enhanced the magnitude of HIV-1-specific CD4+and CD8+T-cell memory immune responses. Splenocytes were collected from mice (n = 4 per group) immunized with DNA-ϕ/MVA-WT, DNA-B/MVA-B or DNA-B/MVA-B ΔA40R 53 days after the last immunization. Next, HIV-1-specific CD4+ and CD8+ T-cell memory immune responses triggered by the different immunization groups were measured by ICS assay as described in the legend to Figure 3. Values from unstimulated controls were subtracted in all cases. *p* values indicate significant response differences between the DNA-B/MVA-B ΔA40R and DNA-B/MVA-B immunization groups (***, *p* < 0.001). Data are from one experiment representative of two independent experiments. (**A**) Overall percentages of HIV-1-specific CD4+ and CD8+ T cells. The values represent the sum of the percentages of T cells expressing CD107a and/or IFN-γ and/or TNF-α and/or IL-2 against Env, Gag, and GPN peptide pools. (**B**) Percentages of Env, Gag, and GPN HIV-1-specific CD4+ and CD8+ T cells. Frequencies represent the sum of the percentages of T cells expressing CD107a and/or IFN-γ and/or TNF-α and/or IL-2 against Env, Gag, or GPN peptide pools. (**C**,**D**) Polyfunctional profiles of HIV-1-specific CD4+ (**C**) and CD8+ (**D**) T cells. All of the possible combinations of responses are shown on the x axis, while the percentages of T cells expressing CD107a and/or IFN-γ and/or TNF-α and/or IL-2 against Env, Gag, and GPN peptide pools are shown on the y axis. Responses are grouped and color-coded on the basis of the number of functions (4, 3, 2, or 1). The pie charts summarize the data. Each slice corresponds to the proportion of the total HIV-1-specific CD4+ and CD8+ T cells exhibiting 1, 2, 3, or 4 functions (CD107a and/or IFN-γ and/or TNF-α and/or IL-2) within the total HIV-1-specific CD4+ and CD8+ T cells.

Similar to the results obtained in the adaptive phase, the magnitude of the total HIV-1-specific CD4+ and CD8+ T-cell memory immune response was again significantly greater in the DNA-B/MVA-B ΔA40R immunization group than in DNA-B/MVA-B (2- and 2-fold higher, respectively), with both vaccinated groups triggering an overall HIV-1-specific immune response mediated mainly by CD8+ T cells (Figure 4A). 

The pattern of HIV-1-specific T-cell memory immune responses showed that CD4+ and CD8+ T cell responses were directed mainly against the Env pool in both vaccinated groups, with both CD4+ and CD8+ T-cell responses broadly distributed among Env, Gag, and GPN (Figure 4B). However, DNA-B/MVA-B ΔA40R significantly enhanced the magnitude of Env- and GPN-specific CD4+ T-cell memory responses and Env-, Gag- and GPN-specific CD8+ T-cell memory responses (Figure 4B).

The quality of the HIV-1-specific CD4+ and CD8+ T-cell memory immune responses was characterized as described above (Figure 4C,D). HIV-1-specific CD4+ T-cell memory responses were more polyfunctional in the group immunized with DNA-B/MVA-B ΔA40R than in DNA-B/MVA-B, with 77% and 49% of the CD4+ T cells exhibiting two or more functions, respectively (Figure 4C, pie charts). CD4+ T cells expressing CD107a-IFN-γ-TNF-α-IL-2, IFN-γ-TNF-α-IL-2, and CD107a-IFN-γ-TNF-α were the most induced populations elicited by both vaccinated groups, with DNA-B/MVA-B ΔA40R inducing a significantly greater percentage of these populations (Figure 4C, bars). On the other hand, as shown in Figure 4D (pie charts), HIV-1-specific CD8+ T-cell memory responses were also more polyfunctional in the group immunized with DNA-B/MVA-B ΔA40R than in DNA-B/MVA-B, with 93% and 88% of the CD8+ T cells exhibiting two or more functions, respectively. CD8+T cells expressing CD107a-IFN-γ-TNF-α-IL-2, CD107a-IFN-γ-TNF-α and CD107a-IFN-γ were the most abundant populations elicited by both vaccinated groups, with DNA-B/MVA-B ΔA40R, once again, inducing a significantly greater increase in the percentage of those populations than DNA-B/MVA-B (Figure 4D, bars). 

### 3.6. MVA-B ΔA40R Enhanced HIV-1-Specific T Cells with an Effector Memory Phenotype in the Adaptive and Memory Phases

**Figure 5:**
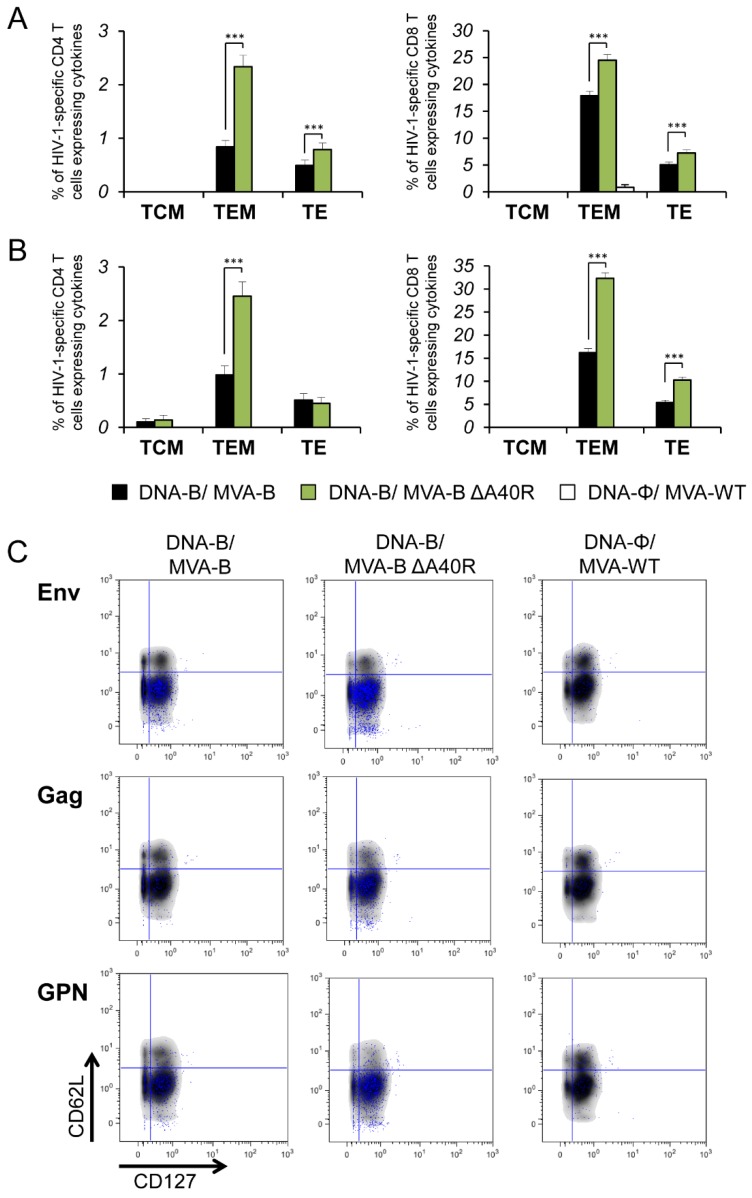
Phenotypic profile of adaptive and memory HIV-1-specific CD4+ and CD8+ T cells. Percentages of TCM, TEM, and TE HIV-1-specific CD4+ and CD8+ T cells expressing CD107a and/or IFN-γ and/or TNF-α and/or IL-2 against Env, Gag, and GPN peptide pools in the adaptive (**A**) and memory (**B**) phases. Values from unstimulated controls were subtracted in all cases. *p* values indicate significant response differences between the DNA-B/MVA-B ΔA40R and DNA-B/MVA-B immunization groups (***, *p* < 0.001). (**C**) Representative flow cytometry phenotypic profile plots of memory HIV-1-specific CD8+ T-cell responses against Env, Gag, and GPN peptide pools. CD8+ T cells expressing CD127 and/or CD62L are shown as density plots in grey and blue dots representing Env-, Gag-, and/or GPN-specific CD8+ T cells expressing CD107a and/or IFN-γ and/or TNF-α and/or IL-2.

It has been described that HIV-1-specific T cells with a mature effector memory phenotype are more frequently detectable in HIV-1 controllers than in HIV-1 progressors [61,62,63]. Thus, we next determined the phenotype of the adaptive and memory HIV-1-specific CD4+ and CD8+ T cells by measuring the expression of the CD127 and CD62L surface markers, which allowed the definition of the different memory subpopulations: T central memory (TCM, CD127^+^/CD62L^+^), T effector memory (TEM, CD127^+^/CD62L^−^), and T effector (TE, CD127^−^/CD62L^−^) cells [64], and determined the sums of the individual responses expressing CD107a, IFN-γ, TNF-α, and/or IL-2 obtained for the Env, Gag, and GPN peptide pools (Figure 5). The results showed that in both vaccinated groups, adaptive and memory HIV-1-specific CD4+ and CD8+ T cells were mainly of the TEM phenotype, followed by the TE phenotype. However, immunization with DNA-B/MVA-B ΔA40R induced a significant increase in the percentage of adaptive and memory HIV-1-specific CD4+ and CD8+ TEM and TE cells (Figure 5A,B). Representative flow cytometry plots of memory HIV-1-specific CD8+ T cells against Env, Gag, and GPN peptide pools are shown in Figure 5C.

### 3.7. MVA-B ΔA40R Increased the Levels of Binding IgG Antibodies Against HIV-1 gp120 

**Figure 6:**
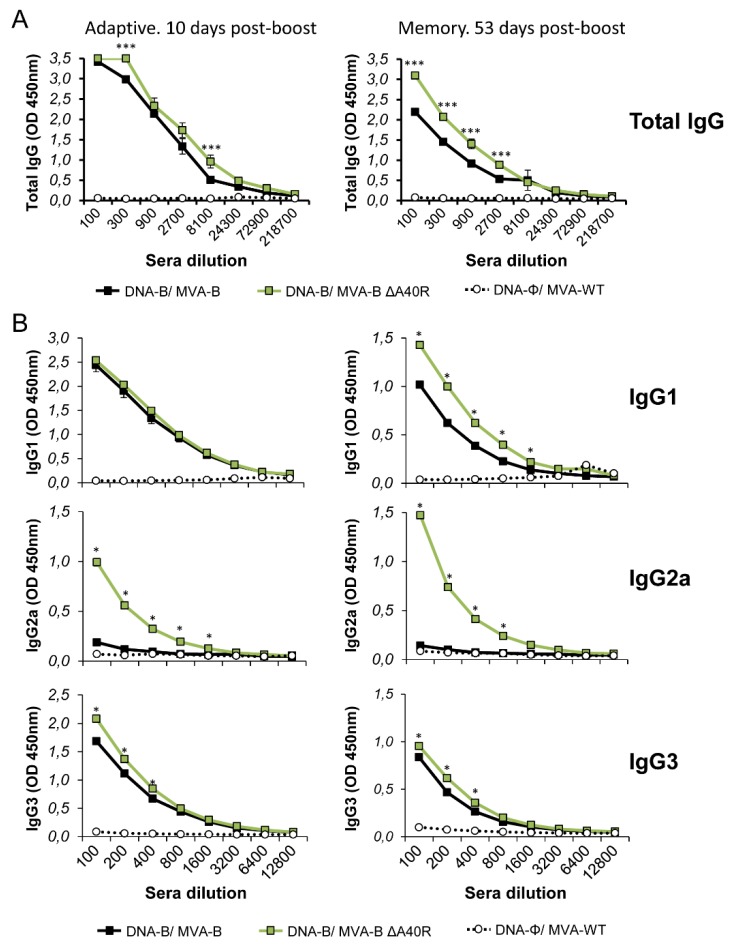
Humoral immune responses elicited by MVA-B and MVA-B ΔA40R against HIV-1 gp120 protein. Levels of gp120-specific total IgG (**A**), and isotype IgG1, IgG2a, and IgG3 (**B**) binding antibodies were measured by ELISA in pooled sera from mice immunized with DNA-B/MVA-B, DNA-B/MVA-B ΔA40R B or DNA-ϕ/MVA-WT (n = 4, at each time point) 10 days (left panels) or 53 days (right panels) after the last immunization. Mean absorbance values (measured at 450 nm) and standard deviations of duplicate pooled serum dilutions are presented. *p* values indicate significant differences in antibody levels between the DNA-B/MVA-B and DNA-B/MVA-B ΔA40R immunization groups (*, *p* < 0.05; ***, *p* < 0.001) at each dilution. Data are from one experiment representative of at least two independent experiments.

Since MVA-B is able to release monomeric gp120 from infected cells and induce humoral immune responses [16], which are thought to be necessary to control HIV-1 infection [65], we next analyzed the humoral immune responses elicited after immunization of mice with DNA-B/MVA-B and DNA-B/MVA-B ΔA40R. Thus, we quantified by ELISA the total IgG and subclass IgG1, IgG2a, and IgG3 levels of antibodies against HIV-1 gp120 (clade B, isolate BX08) in pooled sera obtained from mice 10 and 53 days post-boost (Figure 6). The results showed that DNA-B/MVA-B ΔA40R elicited significantly higher levels of total IgG anti-gp120 antibodies than DNA-B/MVA-B in the adaptive and memory phases (Figure 6A). Furthermore, analysis of the IgG subtypes showed that DNA-B/MVA-B ΔA40R induced significantly higher levels of IgG1, IgG2a, and IgG3 anti-gp120 antibodies than DNA-B/MVA-B (Figure 6B), with IgG1 levels being higher than IgG3 and IgG2a levels, in both groups indicating a Th2 response. However, the analysis of the IgG2a/IgG1 ratio showed that DNA-B/MVA-B ΔA40R induced a higher ratio than DNA-B/MVA-B, suggesting a shift toward a Th1 response. 

### 3.8. Generation of a Revertant MVA-B ΔA40R-rev Virus Expressing High Levels of MVA A40 Protein

To further confirm the immunosuppressive role of the MVA A40 protein, a revertant virus termed MVA-B ΔA40R-rev was constructed by reinserting the MVA *A40R* gene into the MVA HA locus (VACV *A56R* gene) of the MVA-B ΔA40R virus, where the gene was expressed under the control of the VACV synthetic early/late (sE/L) promoter. We selected this synthetic promoter due to the inability to detect the A40 protein expression by Western blot and confocal immunofluorescence, from its own promoter in MVA-infected cells using two different anti-A40 antibodies (generously provided by Drs Geoffrey L. Smith and Jacomine Krijnse-Locker). 

**Figure 7:**
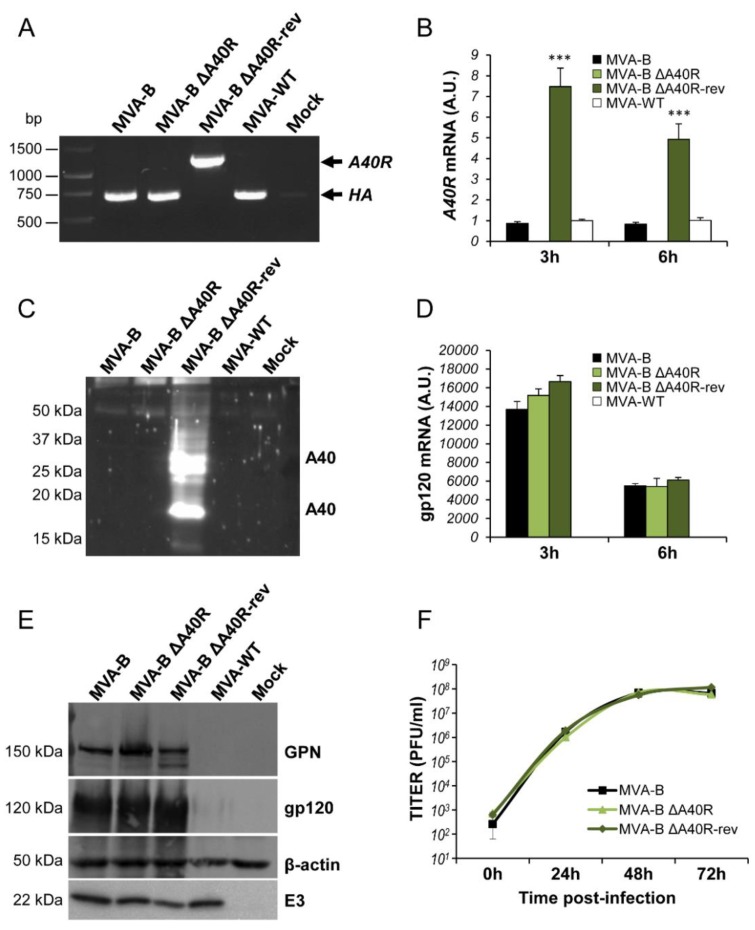
Generation and in vitro characterization of MVA-B ΔA40R-rev virus. (**A**) PCR analysis of the HA locus. Viral DNA was extracted from DF-1 cells mock infected or infected at 5 PFU/cell with MVA-WT, MVA-B, MVA-B ΔA40R, or MVA-B ΔA40R-rev. Primers spanning the HA flanking regions were used for PCR analysis of the HA locus. DNA products corresponding to the WT HA gene (HA) or to the *A40R* gene inserted in the HA locus (*A40R*) are indicated on the right. Molecular size markers (1 kb ladder) with the corresponding sizes (base pairs) are indicated on the left. (**B**) mRNA levels of MVA *A40R* gene. Human THP-1 macrophages were mock infected or infected with MVA-WT, MVA-B, MVA-B ΔA40R, or MVA-B ΔA40R-rev at 5 PFU/cell. At 3 and 6 h.p.i., RNA was extracted, and MVA *A40R* and VACV *E3L* mRNA levels were analyzed by RT-qPCR. Results are expressed as the ratio of the MVA *A40R* gene to VACV *E3L* mRNA levels. A.U.: arbitrary units. *p* values indicate significant response differences between the different viruses at the same hour (***, *p* < 0.001). Data are means ± standard deviations of duplicate samples from one experiment and are representative of two independent experiments. (**C**) Expression of MVA A40 protein. DF-1 cells were mock infected or infected at 5 PFU/cell with MVA-WT, MVA-B, MVA-B ΔA40R, or MVA-B ΔA40R-rev. At 24 h.p.i. cells were lysed in Laemmli buffer, fractionated by 8% SDS-PAGE, and analyzed by Western blotting with rabbit polyclonal anti-A40 antibody. On the right is indicated the position of the MVA A40 protein. The sizes (in kDa) of standards (Precision Plus protein standards; Bio-Rad Laboratories) are indicated on the left. (**D**) mRNA levels of HIV-1_BX08_ gp120 gene. Human THP-1 macrophages were mock infected or infected with MVA-WT, MVA-B, MVA-B ΔA40R, or MVA-B ΔA40R-rev at 5 PFU/cell. At 3 and 6 h.p.i., RNA was extracted, and HIV-1_BX08_ gp120 and endogenous HPRT mRNA levels were analyzed by RT-qPCR. Results are expressed as the ratio of the HIV-1_BX08_ gp120 gene to HPRT mRNA levels. A.U.: arbitrary units. Data are means ± standard deviations of duplicate samples from one experiment and are representative of two independent experiments. (**E**) Expression of HIV-1_BX08_ gp120 and HIV-1_IIIB_GPN proteins. DF-1 cells were mock infected or infected at 5 PFU/cell with MVA-WT, MVA-B, MVA-B ΔA40R, or MVA-B ΔA40R-rev. At 24 h.p.i., cells were lysed in Laemmli buffer, fractionated by 8% SDS-PAGE, and analyzed by Western blotting with rabbit polyclonal anti-gp120 antibody or rabbit anti-gag p24 serum. Rabbit anti-β-actin and rabbit anti-VACV early E3 protein antibodies were used as protein and VACV loading controls, respectively. The proteins detected are indicated on the right and their protein molecular weights (in kDa) are indicated on the left. (**F**) Viral growth kinetics. DF-1 cells were infected at 0.01 PFU/cell with MVA-B, MVA-B ΔA40R, or MVA-B ΔA40R-rev. At different times (0, 24, 48, and 72 h.p.i.) cells were collected and virus titers of cell lysates were quantified by plaque immunostaining assay with anti-VACV antibodies. The mean and standard deviations of two independent experiments is shown.

PCR analysis with primers annealing within the HA flanking regions showed the correct reintroduction of the *A40R* gene in MVA-B ΔA40R (Figure 7A), further confirmed by DNA sequencing. Next, the study of the MVA *A40R* mRNA expression by RT-qPCR confirmed that while the deletion mutant MVA-B ΔA40R did not express *A40R* mRNA, MVA-WT, MVA-B and revertant MVA-B ΔA40R-rev viruses expressed MVA *A40R* mRNA (Figure 7B). Interestingly, MVA-B ΔA40R-rev expressed the highest levels of MVA *A40R* mRNA (7.5- and 5-fold higher than MVA-WT or MVA-B, at 3 and 6 hours post-infection (h.p.i.), respectively) (Figure 7B), probably due to the stronger effect of the VACV sE/L promoter in comparison with the natural *A40R* virus promoter. The higher expression levels of MVA *A40R* mRNA triggered by MVA-B ΔA40R-rev correlated with higher levels of MVA A40 protein (Figure 7C). The presence of two bands in the Western blot using the anti-A40 antibody was compatible with different post-translational modifications, the upper band being compatible with the full-length glycosylated A40 protein. Expression of A40 protein from cells infected with MVA-B or MVA-WT was not detected by Western blotting with the two antibodies used, indicating low levels of A40 protein expression under the regulation of its natural promoter.

Moreover, expression analysis by RT-qPCR and Western blotting showed that MVA-B ΔA40R-rev expressed similar levels of HIV-1_BX08_ gp120 mRNA (Figure 7D) and HIV-1_BX08_ gp120 and HIV-1_IIIB_ GPN proteins (Figure 7E) to MVA-B and MVA-B ΔA40R, confirming that the expression of HIV-1 antigens was not impaired because of the reintroduction and overexpression of the MVA *A40R* gene. 

Finally, the growth kinetics of MVA-B ΔA40R-rev in cultured permissive DF-1 cells was similar to that of MVA-B and MVA-B ΔA40R, confirming that the reintroduction of the MVA *A40R* gene did not affect MVA replication in vitro (Figure 7F).

### 3.9. MVA A40 Protein Localized at the Cell Membrane 

**Figure 8:**
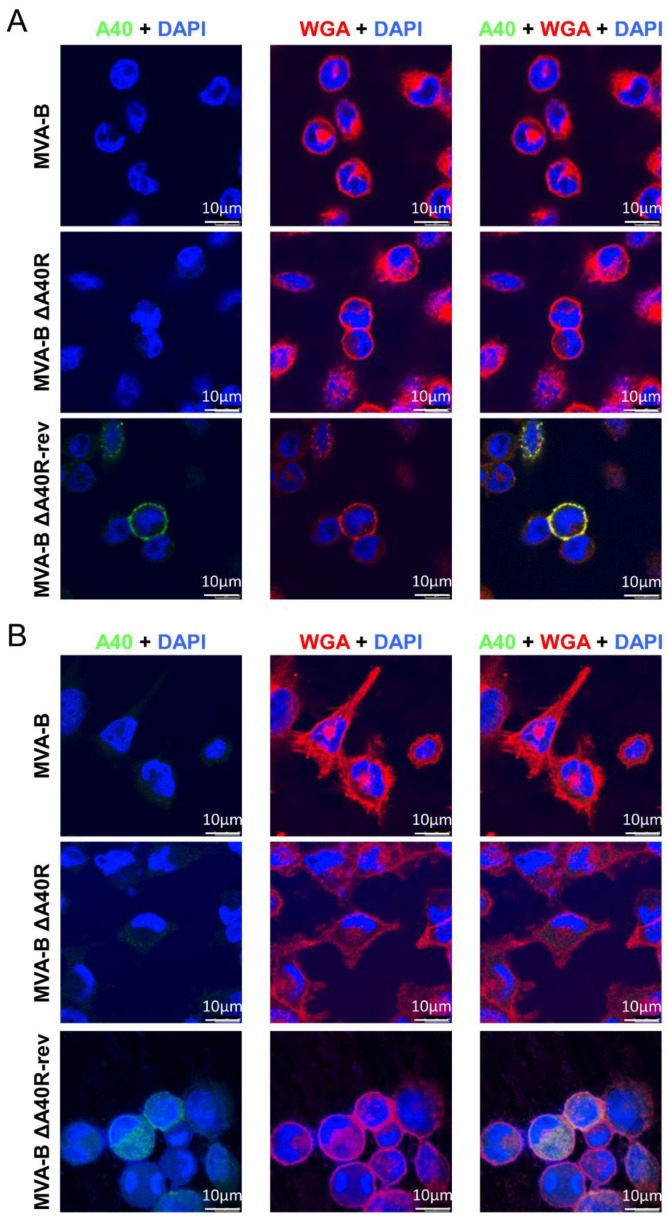
MVA A40 protein localized at the cell membrane. HeLa cells were infected at 0.5 PFU/cell with MVA-B, MVA-B ΔA40R, and MVA-B ΔA40R-rev, and at 18 h.p.i. non-permeabilized (**A**) or permeabilized (**B**) fixed cells were stained with a rabbit anti-A40 polyclonal antibody further detected with an anti-rabbit secondary antibody conjugated with the fluorochrome Alexa Fluor 488 (green) and with a wheat germ agglutinin (WGA) probe conjugated to the fluorescent dye Alexa Fluor 594 (red). Cell nuclei were stained using 4′,6-diamidino-2-phenylindole (DAPI) (blue). Scale bar: 10 μm.

Previous studies have reported that VACV WR A40 protein is expressed at the cell surface [44]. Thus, the expression and intracellular localization of the MVA A40 protein expressed by MVA-B ΔA40R-rev was next studied by confocal immunofluorescence microscopy in non-permissive HeLa cells. Therefore, cells were infected with MVA-B, MVA-B ΔA40R, and MVA-B ΔA40R-rev for 18 h, and then non-permeabilized or permeabilized fixed cells were stained with a polyclonal antibody against VACV A40 protein and the specific wheat germ agglutinin (WGA) probe to label the cell surface and Golgi reticulum (Figure 8). The results showed that in non-permeabilized cells, A40 protein (in green) was expressed in cells infected with MVA-B ΔA40R-rev and, as expected, co-localized with the cell membrane, whereas it was not detected in cells infected with MVA-B or MVA-B ΔA40R (Figure 8A). On the other hand, in permeabilized cells, A40 protein (in green) was expressed in cells infected with MVA-B ΔA40R-rev with a diffused cytoplasmic pattern; again, no detection of A40 protein was observed in cells infected with MVA-B or MVA-B ΔA40R (Figure 8B), probably due to poor reactivity of the anti-A40 antibody used.

### 3.10. Reintroduction of MVA A40R Gene in MVA-B ΔA40R Inhibited Innate Immune Responses In Vitro

**Figure 9:**
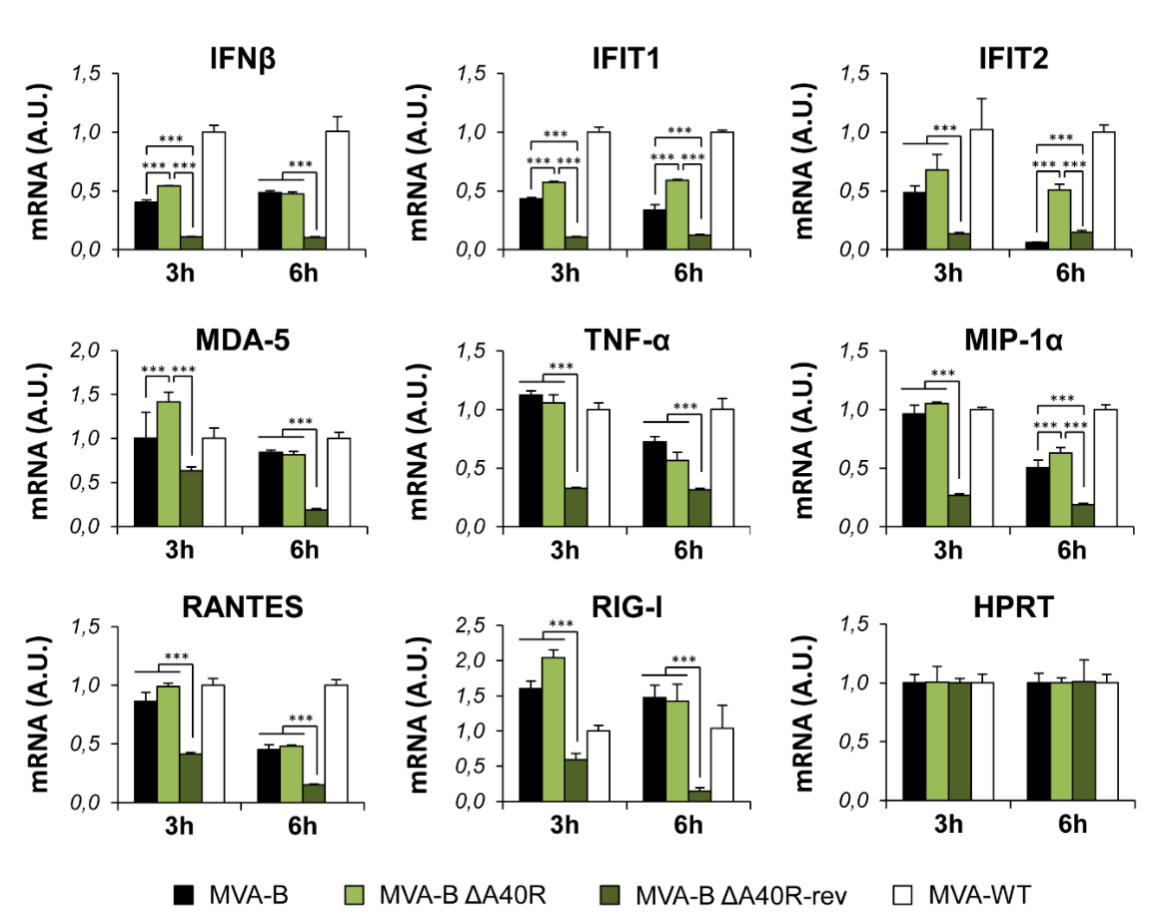
MVA-B ΔA40R-rev downregulated the mRNA levels of type I IFN, proinflammatory cytokines, and chemokines. Human THP-1 macrophages were mock infected or infected with MVA-WT, MVA-B, MVA-B ΔA40R, or MVA-B ΔA40R-rev at 5 PFU/cell. At 3 and 6 h.p.i., RNA was extracted, and IFN-β, IFIT1, IFIT2, MDA-5, TNF-α, MIP-1α, RANTES, RIG-I, HPRT, and VACV *E3L* mRNA levels were analyzed by RT-qPCR. Results are expressed as the ratio of the gene of interest to VACV *E3L* mRNA levels. A.U.: arbitrary units. *p* values indicate significant response differences between MVA-B, MVA-B ΔA40R, and MVA-B ΔA40R-rev at the same hour (**, *p* < 0.005; ***, *p* < 0.001). Data are means ± standard deviations of triplicate samples from one experiment and are representative of two independent experiments.

Next, to confirm whether the reintroduction of MVA *A40R* gene in MVA-B ΔA40R restored the previous enhancement in type I IFN innate immune responses (Figure 2) and to further demonstrate the immunosuppressive role of the MVA A40 protein, we infected human THP-1 macrophages for 3 and 6 h with MVA-WT, MVA-B, MVA-B ΔA40R, and MVA-B ΔA40R-rev at 5 PFU/cell, and analyzed by RT-qPCR the mRNA expression levels of several innate immune-related genes (Figure 9). Interestingly, the results showed that, compared to parental MVA-B ΔA40R and MVA-B, MVA-B ΔA40R-rev significantly downregulated the mRNA levels of IFN-β, IFIT1, IFIT2, MDA-5, RIG-I, and MIP-1α (Figure 9). Moreover, MVA-B ΔA40R-rev downregulated the mRNA levels of other genes not affected by the deletion of MVA *A40R* gene in MVA-B ΔA40R, such as TNF-α and RANTES, while it did not affect the mRNA expression of the endogenous cellular gene HPRT (Figure 9). These results confirm the immunosuppressive role of the MVA A40 protein.

### 3.11. Reintroduction of MVA A40R Gene in MVA-B ΔA40R Impaired HIV-1-Specific T-Cell Immune Responses

**Figure 10:**
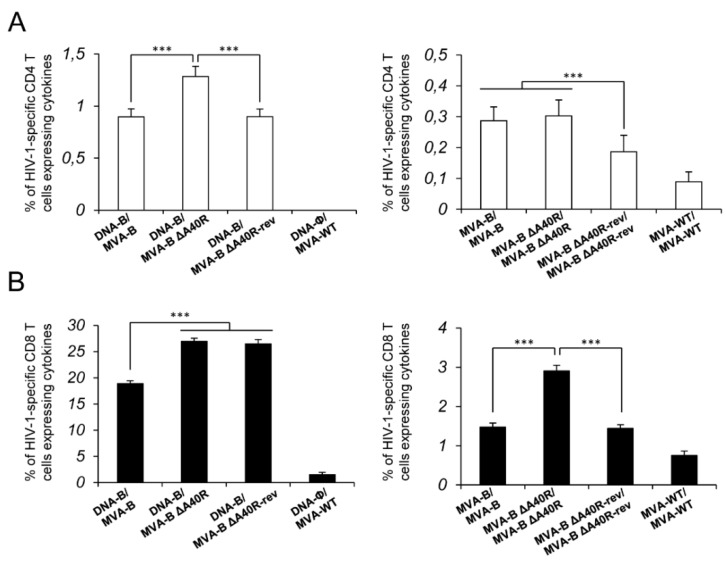
Immunization with MVA-B ΔA40R-rev impairs the magnitude of HIV-1-specific T-cell immune responses. Splenocytes were collected from mice (n = 4 per group) immunized with heterologous DNA/MVA (left panels) or homologous MVA/MVA (right panels) prime/boost immunization protocols, 10 days after the last immunization. Next, HIV-1-specific CD4+ and CD8+ T-cell adaptive immune responses triggered by the different immunization groups were measured by ICS assay as described in the legend to Figure 3. Values from unstimulated controls were subtracted in all cases. *p* values indicate significant response differences between immunization groups (***, *p* < 0.001). (**A**) Overall percentages of HIV-1-specific CD4+ T cells in the DNA/MVA (left panel) or MVA/MVA (right panel) immunization regimens. The values represent the sum of the percentages of CD4+ T cells expressing CD107a and/or IFN-γ and/or TNF-α and/or IL-2 against Env, Gag, and GPN peptide pools. (**B**) Overall percentages of HIV-1-specific CD8+ T cells in the DNA/MVA (left panel) or MVA/MVA (right panel) immunization regimens. The values represent the sum of the percentages of CD8+ T cells expressing CD107a and/or IFN-γ and/or TNF-α and/or IL-2 against Env, Gag, and GPN peptide pools.

Next, to ascertain whether the reintroduction of MVA *A40R* gene in MVA-B ΔA40R could restore HIV-1-specific T-cell immunogenicity to levels similar to those induced by parental MVA-B containing the *A40R* gene (see Figure 3 and Figure 4), we analyzed at 10 days after the last immunization the HIV-1-specific CD4+ and CD8+ T-cell immune responses induced by MVA-B ΔA40R-rev in mice immunized following heterologous DNA/MVA and homologous MVA/MVA prime/boost regimens (Figure 10).

The magnitude of the total HIV-1-specific CD4+ T-cell immune responses induced by the DNA-B/MVA-B ΔA40R-rev (Figure 10A, left panel), or the MVA-B ΔA40R-rev/MVA-B ΔA40R-rev (Figure 10A, right panel) immunization groups was significantly lower than that induced by DNA-B/MVA-B ΔA40R or MVA-B ΔA40R/MVA-B ΔA40R, respectively, but similar to that induced by the DNA-B/MVA-B group and lower than that induced by the MVA-B/MVA-B group (Figure 10A). Furthermore, the analysis of the HIV-1-specific CD8+ T-cell immune responses showed that homologous MVA-B ΔA40R-rev/MVA-B ΔA40R-rev immunization group induced significantly lower responses than MVA-B ΔA40R/MVA-B ΔA40R that were similar to MVA-B/MVA-B (Figure 10B, right panel). However, the heterologous DNA-B/MVA-B ΔA40R-rev immunization group did not decrease the magnitude of HIV-1-specific CD8+ T-cell immune responses compared to DNA-B/MVA-B ΔA40R, probably influenced by the DNA priming that frames the quality of the immune responses prior to a poxvirus protein boost, as recently described [66]. Collectively, these results confirmed that the reintroduction of MVA *A40R* gene in MVA-B ΔA40R restores the magnitude of the HIV-1-specific T-cell immune responses.

## 4. Discussion

Many recombinant poxvirus vectors [such as MVA, New York vaccinia virus (NYVAC), canarypox, and fowlpox viruses] expressing different HIV-1 antigens have been widely used in several human clinical trials in the last few years, proving that they are safe and immunogenic, inducing HIV-1-specific humoral and cellular immune responses [12,67,68]. In fact, the canarypox ALVAC combined with HIV-1 gp120 protein is actually the only effective HIV/AIDS vaccine candidate, and it showed a 31.2% protective effect in a phase III clinical trial [9]. However, this efficacy is modest, and the immunogenicity against HIV-1 antigens induced by modified poxvirus vector-based vaccines tested in human clinical trials is limited. Thus, more efficient and optimized poxvirus vector-based HIV/AIDS vaccines able to enhance HIV-1-specific humoral and cellular immunogenicity are needed [35].

Among the different approaches developed to enhance the immune response induced by poxvirus vectors, one promising strategy is the removal of the VACV genes that antagonize the immune system, as the virus genome still contains several genes that interfere with host immune responses [39,40]. Several recombinant MVA vectors expressing HIV-1 antigens and containing deletions in different immunomodulatory VACV genes have been generated, and have been shown to be able to enhance immune responses to HIV-1 antigens in animal models [18,20,21,23,69,70,71]. 

In this work we described the immunosuppressive function of MVA *A40R* gene and tested its role on antigen-specific immune responses in vivo after deleting the MVA *A40R* gene in the vector backbone of MVA-B, an HIV/AIDS vaccine candidate that expresses HIV-1_IIIB_ GPN as an intracellular polyprotein and HIV-1_BX08_ gp120 as a cell-released product from HIV-1 clade B isolate [16]. Previously to this work, the role of the VACV gene *A40R* was controversial. On one hand, it has been proposed that the VACV gene *A40R* encodes a type II membrane glycoprotein that is expressed early during infection on the cell surface, but is not incorporated into IMV or EEV particles [44]. These authors also showed that the A40 protein shares amino acid similarity with the CDR domain of C-type lectins including Clr-b, natural killer cell receptors, the human IgE receptor, and CD69, an early activation marker on lymphocytes. These C-type lectins are key players in pathogen recognition and innate immunity [45] and, in this regard, the A40 protein might have a role in interfering with the host response to infection. Moreover, the localization of A40 protein at the cell surface suggests that it may modulate the immune response by interacting at the plasma membrane level with signaling pathways and/or with other cells, but there is no evidence for any of these interactions. Interestingly, deletion of the VACV *A40R* gene from the VACV strain WR resulted in a modest attenuation after intradermal inoculation of mice, which could perhaps reflect an immunomodulatory role for this protein [46]. On the other hand, other reports have affirmed that VACV A40 is an early protein that is partially SUMO-1-modified and associated with the viral “mini-nuclei” [48]. Although the small amount of non-sumoylated A40 protein has a role in the VACV life cycle, joining the cytosolic side of the ER and inducing the proper apposition of several ER cisternae before its fusion to generate the ER envelope that surrounds the viral replication sites, the role that sumoylated A40 protein could play in the VACV life cycle still remains unknown; possibly it could participate in the process of replication itself or in the late transcription that happens at VACV replication sites, making A40 essential for VACV life cycle [47]. However, nothing was previously known about the immune function of this VACV gene. 

Consequently, to evaluate whether the VACV A40 protein has an immunomodulatory role, an MVA recombinant vector lacking the MVA *A40R* gene was generated from the HIV/AIDS vaccine candidate MVA-B (termed MVA-B ΔA40R). It is of note that the amino acid sequence of WR A40 protein differs from MVA A40 protein, in which the last five amino acids at the C-terminus are substituted with another 14 unrelated ones. The results showed that *A40R* deletion had no effect on virus growth, demonstrating that MVA *A40R* gene is not essential for VACV life cycle, in contrast to what has been suggested by others [47]. As the loss of immunomodulatory genes in the MVA backbone impairs the innate immune response to this vector [18,20,23,72], the first step to elucidating the supposed immunomodulatory role of the MVA *A40R* gene was to study the innate immune responses triggered in THP-1 human macrophages infected with MVA-WT, parental MVA-B, and MVA-B ΔA40R deletion mutant. The results showed that, compared to parental MVA-B, MVA-B ΔA40R significantly enhanced the expression of several genes involved in the type I IFN signaling pathway, such as IFN-β, IFIT1, and IFIT2, as well as the viral dsRNA sensor MDA-5, and the pro-inflammatory chemokine MIP-1α, suggesting that MVA A40 protein could have an immunomodulatory role, blocking innate immune responses during virus infection. The enhanced levels of these mRNAs in MVA-WT versus MVA-B were likely due to a suppressive effect mediated by expression of the HIV-1 antigens. Moreover, since it has been described that innate immune responses play a critical role in the control and resolution of HIV-1 infection, providing signals for the efficient priming of the adaptive branch of immune response [73], the enhanced IFN-signaling could be an advantage of this MVA-B ΔA40R recombinant vector as HIV/AIDS vaccine candidate. 

To further define whether MVA A40 could impair the immune system in vivo, a DNA prime/MVA boost immunization protocol was performed in mice to compare adaptive and memory immune responses to HIV-1 antigens induced by parental MVA-B and the deletion mutant MVA-B ΔA40R. Results showed that the DNA-B/MVA-B ΔA40R immunization group presented significantly enhanced magnitude of the overall adaptive and memory HIV-1-specific CD4+ and CD8+ T cells expressing CD107a, IFN-γ, TNF-α, and/or IL-2 compared to DNA-B/MVA-B. These results further suggest the immunosuppressive role of MVA A40 protein, as deletions of well-known immunosuppressive VACV genes from MVA or NYVAC vectors expressing HIV-1 antigens have produced similar results [18,20,21,23,69,70,71,74,75,76,77,78]. Furthermore, both immunization groups elicited an adaptive and memory HIV-1-specific T-cell immune response with a similar polyfunctional profile, and mainly TEM and to a lesser extent TE phenotypes. However, once again, MVA-B ΔA40R significantly enhanced the magnitude of these populations, an important and relevant feature because the presence of TEM has been correlated with protection in the macaque SIV model [79,80]. The fast acquisition of TEM and TE phenotypes in the adaptive phase could be important in the development of the T-cell memory responses and in the mounting of a more effective immunity during a primary pathogen encounter. Moreover, adaptive and memory CD4+ T cell immune responses were directed mainly against Env in both immunization groups. However, in contrast to other MVA-B deletion mutants previously characterized (with deletions in *C6L*, *C6L*-*K7R*, and *A41L*-*B16R* MVA genes), where a pattern of GPN-specific CD8+ T-cell immune responses was mainly induced [18,20,21], MVA-B ΔA40R triggered CD8+ T-cell immune responses preferentially directed against Env, similarly to the MVA-B deletion mutant lacking the N2L gene, which encodes for a nuclear inhibitor of IRF3 [23]. The biological relevance of this T cell immune shift is not known. 

The analysis of the gp120-specific humoral immune responses at the adaptive and memory phases showed that DNA-B/MVA-B ΔA40R immunization induced higher levels of total IgG, IgG1, IgG2a, and IgG3 anti-gp120 antibodies than DNA-B/MVA-B. This enhancement in antibodies against gp120 may have been mediated by the increase in innate immune responses and HIV-1-specific CD4+ T helper cells triggered by the MVA-B ΔA40R deletion mutant, and it could be a positive immune parameter, as it has been described that anti-HIV-1 V2-specific IgG1 or IgG3 antibodies are able to drive ADCC correlated with efficacy in the RV144 phase III clinical trial [81].

Moreover, evaluation of the Th1 or Th2 response by analyzing the ratio of IgG2a/IgG1 or IgG3/IgG1 antibodies showed that all the viruses induced higher levels of IgG1 than IgG2a or IgG3 antibodies, indicating a Th2 response. However, MVA-B ΔA40R induced in the adaptive and memory phases a ratio of IgG2a/IgG1 significantly higher than MVA-B (i.e., in the memory phase: 0.16 vs. 0.73 for MVA-B and MVA-B ΔA40R, respectively, at a dilution of 1/200), indicating that MVA-B ΔA40R may induce a more pronounced shift towards a Th1 response than MVA-B. According to current thinking, any therapeutic vaccination approach against HIV-1 should stimulate the induction of cytotoxic T lymphocytes (CTLs) and Th1 cells. It has been demonstrated that Th1 cells are more resistant to HIV-1 replication than Th2 cells [82,83,84], and HIV-1 has evolved to subvert a “protective” Th1 response to a “permissible” Th2/Th0-type immune response [85,86,87]. For example, ALVAC-specific CD4+ T cells from RV144 vaccinees that show protection against HIV-1 infection display a polarized Th1-like phenotype shown to be less susceptible to HIV-1 infection [88], which could positively influence the efficacy of the RV144 trial.

To finally demonstrate that the in vivo effects triggered by MVA-B ΔA40R in immunized mice were due to the deletion of the MVA *A40R* gene, and to confirm our previously suggested immunosuppressive role exerted by the MVA *A40R* gene, a revertant virus termed MVA-B ΔA40R-rev was generated. Due to the lack of detection (using Western blot or immunofluorescence assays) of A40 protein when expressed from its natural locus in MVA or MVA-B viruses, we decided to reintroduce the MVA *A40R* gene into the HA locus of the MVA-B ΔA40R under the control of a stronger sE/L viral promoter. A similar strategy was used previously to generate NYVAC-based revertant viruses, which allowed us to demonstrate the important role of NFκB activation by VACV in enhancing neutrophil migration and HIV-1-specific T-cell responses [77,78]. Moreover, it has been reported that insertion of heterologous genes into the MVA HA locus does not affect the expression of neighboring MVA genes and the recombinant viruses generated are functional, indicating that the HA gene region is a suitable insertion site [89]. In fact, we have used the HA locus to insert in the MVA genome heterologous antigens from Leishmania [90] and Ebola virus [42], and this strategy was effective to trigger antigen-specific T cells, humoral immune responses, and protective efficacy.

The MVA-B ΔA40R-rev expressed the MVA *A40R* gene at higher mRNA levels (5–7-fold) than MVA-B or MVA-WT, allowing the amplification of the A40 protein signal during viral infection. In the MVA-B or MVA-WT viruses, the protein levels of A40 were very low since the rabbit polyclonal antibody anti-A40 used could not detect the A40 protein by Western blot or by immunofluorescence, while in MVA-B ΔA40R-rev-infected cells the A40 protein was readily detected. This was confirmed by Western blot analysis with a multiplicity of infection (MOI) of 5 PFU/cell. For these experiments, we used the same antibody that was described in the first report of the A40 protein from the WR strain [44]. The Western blot with this anti-A40 antibody detected three major bands at 18, 28, and 35 kDa. It has been suggested that the 18 kDa form corresponds with the unglycosylated A40 protein, whereas the 28 and 35 kDa forms (together with a 38 kDa form that was not detected here) correspond with the N- and O-linked glycosylated forms of A40 [44]. The expression of A40 by MVA-B ΔA40R-rev under the stronger sE/L virus promoter in place of its own promoter provided the means to follow the subcellular localization of the A40 protein. The immunofluorescence analysis of MVA-B ΔA40R-rev-infected HeLa cells detected the A40 protein at the cell surface in non-permeabilized cells, appearing as punctuate cytoplasmic structures in permeabilized cells that co-localized with the Golgi network and exocytic vesicles. These results were similar to the results obtained previously [44] and reinforced the membrane localization of A40 protein, in contrast to what was stated by another report [48]. Although it could be hypothesised that the amino acid sequence differences at the C-terminal region of VACV WR and MVA A40 proteins might lead to a less stable and functional MVA A40 protein, the inability to detect the MVA A40 protein (either by Western blot or immunofluorescence assays) using antibodies readily recognizing the VACV WR A40 protein [44] was mainly due to the low levels of A40 naturally expressed from the MVA genome. Interestingly, when the MVA *A40R* gene was inserted into the MVA HA locus (*A56R* gene) controlled by a stronger VACV promoter than its naturally occurring promoter, the MVA A40 protein was clearly detectable by Western blot and immunofluorescence assays using the anti-A40 antibody recognizing the VACV WR A40 protein, indicating that the MVA A40 protein was stable, correctly expressed, and detected in the cell membrane, as previously described with the WR A40 protein [44].

Importantly, the RT-qPCR experiments showed that mRNA levels of IFN-β, IFIT1, IFIT2, MDA-5, TNF-α, MIP-1α, RANTES, and RIG-I in MVA-B ΔA40R-rev–infected human THP-1 macrophages were significantly lower than those induced by MVA-B ΔA40R or MVA-B. However, mRNA levels of the constitutive cellular gene, HPRT, and the heterologous HIV-1 gp120 gene were not affected, suggesting that A40 blocks specifically innate immune sensing genes. The clear decrease in the levels of IFN-β, IFN-induced genes, proinflammatory cytokines, and chemokines induced by the revertant virus MVA-B ΔA40R-rev, expressing high mRNA levels of the MVA *A40R* gene and of A40 protein, strongly suggest the immunosuppressive function of A40 by blocking the type I IFN signaling pathway.

Interestingly, the enhancing effect on adaptive HIV-1-specific CD4+ and CD8+ T-cell immune responses observed with MVA-B ΔA40R was restored, in general, to levels similar or lower to those induced by MVA-B when we immunized mice with MVA-B ΔA40R-rev, either in DNA/MVA or in MVA/MVA regimens, further confirming in vivo the immunosuppressive function of MVA A40 protein.

## 5. Conclusions

Overall, our findings revealed that MVA *A40R* gene has an immunosuppressive role, with its deletion enhancing innate immune responses in cell cultures, and adaptive and memory HIV-1-specific CD4+ and CD8+ T-cell and humoral immune responses in vivo, while its overexpression inhibited innate responses in human macrophages and adaptive HIV-1-specific CD4+ and/or CD8+ T-cell immune responses in immunized mice. Thus, deletion of the *A40R* gene has an immunomodulatory role in VACV infection and ablation of this gene provides an important strategy for the optimization of MVA vectors as vaccines.
